# Restless Legs Syndrome and the Use of Antipsychotic Medication: An Updated Literature Review

**DOI:** 10.7759/cureus.27821

**Published:** 2022-08-09

**Authors:** Wael K Saber, Ahad R Almuallim, Rami Algahtani

**Affiliations:** 1 Department of Medicine, Umm AlQura University School of Medicine, Makkah, SAU

**Keywords:** quetiapine, olanzapine, antipsychotics, willis-ekbom disease, restless legs syndrome

## Abstract

Restless legs syndrome or Willis-Ekbom disease (RLS/WED) is a sleep-related movement disorder characterized by an urge to move the legs. This impulse is usually accompanied by an uncomfortable and unpleasant sensation in the legs, which worsens at night and during periods of inactivity and is relieved by movement. Several studies in the literature reported the association between RLS and different antipsychotic medications. with Olanzapine, Quetiapine, and Clozapine identified as the most common causes. The literature suggests that the development of RLS in antipsychotic users may be attributed to the inhibition of dopaminergic neurotransmission or the impact of antipsychotics on iron metabolism. Diagnosing antipsychotic-induced RLS remains a substantial challenge in clinical practice, with challenges in the management of this condition also being widely reported in the current literature. In this article, we will review the evidence suggesting the association between RLS and the use of antipsychotic medications, differentiate between RLS and other movement disorders, and give a brief review of the pathophysiology, diagnosis, and management of RLS and its challenges among psychotic patients.

## Introduction and background

Restless legs syndrome or Willis-Ekbom disease (RLS/WED) is a sleep-related movement disorder characterized by an urge to move the legs. This impulse is usually accompanied by an uncomfortable and unpleasant sensation in the legs, which worsens at night and during periods of inactivity and is relieved by movement [1-2]. The prevalence rate of RLS is 5-15% in the general population, with 2.5% of adults having symptoms severe enough to require medical intervention [3]. However, despite the increasing rates of patients with RLS, many suffer from RLS symptoms without ever getting a medical diagnosis. For example, a study found that 6.2% of those seeking medical attention for symptoms of RLS reported having received an RLS diagnosis [4].

RLS has several untoward health outcomes. For example, a cohort study in 2019 demonstrated a positive association between the risk of suicide and self-harm and RLS [5]. Moreover, a prospective cohort study observed that, within six years of follow-up, women with RLS had a higher risk of developing coronary heart disease [6]. Furthermore, a meta-analysis of five population-based studies indicated the association between RLS and hypertension [7]. RLS has also been linked to obesity in a meta-analytic study in 2018 [8].

Several studies in the literature reported the association between RLS and different antipsychotic medications. For instance, a review article on drug-induced RLS listed antipsychotics as one of the most common drugs suspected of causing RLS, along with antidepressants and antiepileptics [9]. According to multiple reviews and case reports, Olanzapine is the most reported antipsychotic associated with RLS [10-14]. Antipsychotics and RLS have been linked since 1999, after a case report suggested that Olanzapine may cause RLS [13]. Furthermore, a Korean study in 2007 reported a significantly higher prevalence of RLS in schizophrenic patients undergoing antipsychotic treatment (21.4%) compared to the control group (9.3%) with a p-value of 0.009 [15]. Additionally, a German study demonstrated the prevalence of RLS among psychiatric inpatients to be 16.4%, 76.9% of whom were diagnosed for the first time [16].

In this article, we will review the evidence suggesting the association between RLS and the use of antipsychotic medications, differentiate between RLS and other movement disorders, and give a brief review of the pathophysiology, diagnosis, and management of RLS and its challenges among psychotic patients.

Methodology

The objectives of this literature review were centered around the hypothesis that antipsychotic medications increase the risk of RLS development. Therefore, the primary objective was to assess the underlying factors that may contribute to this enhanced risk. The secondary objectives included differentiating between RLS and other movement disorders, providing an overview of the management of RLS and its challenges among psychotic patients, and deducing any special considerations in treating RLS in antipsychotic medication users.

A comprehensive search of the literature databases PubMed, Medline, and Google Scholar was conducted. A Population/Exposure/Outcome (PEO) framework, as presented in Table 1, was devised to support the literature search and identify the key terms to be incorporated.

**Table 1:** PEO framework PEO: Population/Exposure/Outcome

PEO	Keywords
Population	Restless legs syndrome, Willis-Ekbom disease, RLS, WED.
Exposure	Antipsychotics, antipsychotic medication, Clozapine, Olanzapine, Quetiapine
Outcome	Risk of RLS, RLS, psychosis, challenges, genetic factors

## Review

Pathophysiology and genetic basis

Evidence suggests that genetic factors play a significant role in the etiology of RLS [17]. The Familia studies showed an autosomal dominant inheritance while other data reported an autosomal recessive and non-mendelian inheritance [17]. However, most of the genetic factors related to RLS were related to primary RLS, with little evidence available on secondary RLS and genetic factors [17]. Some people develop secondary RLS when exposed to current causative factors while others do not, indicating a possible genetic reason behind secondary RLS. The relation between the genetic factors and antipsychotics inducing RLS is still unclear; however, a cohort study in Korea suggested that the haplotype MAP2K5 polymorphisms increased the risk of antipsychotics inducing RLS [18]. Another study in Korea also suggested that the haplotype frequencies among the MAOA (monoamine oxidase A) gene VNTR and the MAOA gene A644g were associated with the severity of antipsychotics inducing RLS among schizophrenic patients [19].

Until recently, the pathophysiology of RLS was poorly comprehended. Current research suggests that the pathophysiology of RLS is centered around dopaminergic dysfunction, reduced central nervous system iron, genetic linkages, or alteration in neurotransmitters [20]. Therefore, disturbances in the above homeostasis are expected to cause secondary RLS.

A clear indication of dopamine pathology in RLS was observed in an autopsy study [21]. This research showed a significant reduction in D3 receptors in the putamen nucleus and an abnormally elevated level of tyrosine hydroxylase (TH) in the substantia nigra when compared to the control group but no changes in D1 receptors were noted [21]. It is currently unknown whether the dopamine effect arises as a result of hyper- or hypodopaminergic states within the nervous system and whether the dysfunction originates in the spinal cord or basal ganglia. Yet, the data shows that dysfunction of the D3 receptor, which promotes an inhibitory effect, increases the sensitivity of spinal reflex pathways. The spinal sensorimotor circuits may be the key to the origination of the sensory symptoms of RLS [22].

Antipsychotics’ mechanisms of action rely primarily on the inhibition of dopaminergic neurotransmission, along with multiple other pathways. The dopaminergic inhibition may explain the link between antipsychotics and secondary RLS as demonstrated by other studies [23]. Another possible rationale is the effect of antipsychotics on iron metabolism. A meta-analysis of cross-sectional studies showed that patients who developed akathisia after using antipsychotics have a lower ferritin level [24]. Furthermore, it has also been reported that iron deficiency anemia is induced by antipsychotics, providing further evidence for the relationship between antipsychotics and RLS [23]. However, additional studies are needed to establish the pathophysiological basis of abnormal iron metabolism resulting in antipsychotic-induced RLS.

Classification

RLS is classified according to its underlying pathology and is termed primary or secondary RLS. Primary RLS is associated with an idiopathic cause while secondary RLS has multiple causes such as psychotropic medication, alcohol abuse, pregnancy, and iron deficiency anemia [25]. RLS is further classified according to its age of onset. Patients who develop RLS before 45 years of age are classified as early-onset RLS while others who develop RLS later in life are classified as late-onset RLS [25]. Early-onset RLS has been linked to genetic factors and better outcomes [26].

Diagnosis

RLS is a clinical diagnosis based on a thorough assessment of patient history and a physical examination. There is no confirmative test for the diagnosis of RLS. In 1995 the International Restless Legs Syndrome Study Group (IRLSSG) developed the first diagnostic criteria for RLS, which was revised in 2003 to provide greater reliability and validity [3]. The 2003 revised criteria, referred to as the IRLSSG consensus criteria, contributed to several milestones in the field of RLS. First, it served as an essential component in many epidemiological studies. Second, it aided in clinical trials establishing the first-line treatment for moderate to severe RLS. Finally, it served as a base for discovering multiple aspects of RLS/WED biology [27]. Rapid progress in the field of RLS/WED over the past several years revealed limitations of the 2003 IRLSSG criteria, which were further revised in 2012 to improve the specificity of diagnosis in clinical and research settings. The 2012 revised criteria consist of essential diagnostic and supportive criteria, as evidenced in Tables 2-3 [27-28].

**Table 2:** IRLSSG: International Restless Legs Syndrome Study Group Criteria [27,29]

Essential Criteria
1. An urge to move the legs, usually accompanied or caused by uncomfortable and unpleasant sensations in the legs. Sometimes, the urge to move is present without the uncomfortable sensations, and sometimes the arms or other body parts are involved in addition to the legs.
2. The urge to move or unpleasant sensations begin or worsen during periods of rest or inactivity such as lying or sitting.
3. The urge to move or unpleasant sensations are partially or totally relieved by movement, such as walking or stretching, at least as long as the activity continues.
4. The urge to move or unpleasant sensations are worse in the evening or night than during the day or only occur in the evening or night. When symptoms are severe, the worsening at night may not be noticeable but must have been previously present.
5. Symptoms are not solely accounted for by another medical or behavioral condition such as leg cramps or habitual foot tapping.

**Table 3:** IRLSSG: International Restless Legs Syndrome Study Group Criteria [27,29]

Supportive Criteria
A family history of RLS.
A positive response to dopaminergic drugs.
Periodic limb movements during wakefulness or sleep as assessed with polysomnography or leg activity devices.

Under the initial diagnostic criteria, several conditions were mistaken for RLS, as their presentation mimicked the first four criteria. Hence, the revision of the 2003 criteria aimed to exclude this limitation through the addition of the fifth criteria [27]. The most common conditions that mimic RLS are sleep-related leg cramps, positional leg cramps, venous disorder, polyneuropathy, and akathisia [29]. Akathisia is one of the most common side effects associated with antipsychotics, and it should be ruled out in antipsychotic users before diagnosing RLS [27,30]. In contrast to RLS, akathisia is often described as an inner feeling of restlessness that affects the whole body. The patient may rock back and forth, cross, uncross or shift from one foot to the other [29]. RLS has a localized area of paraesthesia in the limbs, is most active during evening/night, affects sleep, and has no history of exposure to neuroleptic medications or associated extrapyramidal symptoms [31]. In cases of uncertainty, the supportive IRLSSG criteria are helpful in differentiating between the two causes.

The diagnosis of antipsychotics-inducing RLSs can be challenging for a variety of reasons. First, antipsychotics can cause other movement disorders, such as akathisia and parkinsonism, which are common mimickers of RLS [30]. Second, RLS can coexist with other mimickers of RLS, which may delay RLS diagnosis [32]. Third, psychiatric patients are at an increased risk of RLS when compared to the general population; therefore, it makes it more challenging to attribute the cause of RLS to antipsychotic use [16]. Finally, some reports even suggested that RLS may go underrecognized or ignored [33].

Different antipsychotics and RLS

Olanzapine, Quetiapine, and Clozapine are the most common RLS-inducing antipsychotics [34-37]. However, this contradicts the current comprehension of antipsychotics and their RLS-inducing mechanism of action, given the low affinity of quetiapine to D2 receptors. Moreover, it has been observed that, in some cases, changes in antipsychotic use resolve RLS symptoms, which cannot be explained by the dopaminergic theory of RLS [37].

When combined with other medications, antipsychotics induce RLS, with the current literature demonstrating an increased risk of secondary RLS following the use of concomitant medication with antipsychotics [9]. A cross-sectional study suggested that concurrent treatment of antipsychotics with antidepressants is more susceptible to secondary RLS [38]. This is hypothesized to be due to the inhibitory effect of antidepressants on dopamine functions [9]. Another example is verapamil, which acts as an inhibitor of antipsychotic metabolism, hence, increasing the risk of RLS among antipsychotic users [39].

Challenges in RLS management in patients with psychosis

The management of RLS includes iron replacement therapy, pharmacological therapy, and non-pharmacological therapy [40]. General considerations when deducing RLS treatment involve assessing and managing coexisting sleep disorders and the use of medications causing or exacerbating RLS. Typically, the treatment of RLS is divided into three categories according to the chronicity and severity of RLS symptoms. Iron replacement therapy is suggested in RLS patients with a serum ferritin level of ≤ 75 ng/ml and transferrin saturation of < 45% either orally or intravenously [41-43].

For the treatment of intermittent RLS, defined as RLS symptoms that are severe enough to require treatment but occur on average less than twice per week, non-pharmacological therapy is advised. This approach comprises behavioral strategies, including moderate regular exercise, mental alerting activities, and abstinence from caffeine and alcohol [41,44-45]. Levodopa, benzodiazepines, and low-potency opioids (codeine, tramadol) are on-demand medications recommended for intermittent RLS. In the case of chronic persistent RLS, defined as RLS symptoms that are frequent and severe enough to require daily treatment, usually occurring on average at least twice a week and resulting in moderate or severe distress, non-pharmacological therapy is recommended in addition to alpha-2-delta calcium channel ligands (gabapentin, pregabalin) and non-ergot dopamine agonists (pramipexole, ropinirole) if calcium channel ligands are contradicted [41]. Lastly, for refractory RLS, defined as RLS unresponsive to monotherapy with tolerable doses of first-line agents due to reduction in efficacy, augmentation, or adverse effects, combination therapy of different classes of drugs (dopamine agonist, alpha-2-delta ligand, opioid, benzodiazepine) or opioid monotherapy is recommended [41]. Table 4 provides an overview of RLS treatment according to chronicity.

**Table 4:** General treatment of RLS according to chronicity *RLS symptoms that are troublesome enough to require treatment but occur on average less than twice per week **RLS symptoms that are frequent and troublesome enough to require daily treatment, usually occurring on average at least twice a week and resulting in moderate or severe distress ***RLS unresponsive to monotherapy with tolerable doses of first-line agents due to reduction in efficacy, augmentation, or adverse effects RLS: restless legs syndrome [42,44-45]

Intermittent RLS^*^	Chronic RLS^**^	Refractory RLS^***^
General Consideration: Iron replacement therapy in patients with serum ferritin level: ≤ 75 ng/ml, and transferrin saturation: < 45%		
Non-pharmacological therapy: 1-behavioral strategies which include moderate regular exercise and mental alerting activities 2-abstinence from caffeine and alcohol		Pharmacological therapy: combination therapy of different classes of drugs [dopamine agonist, alpha-2-delta ligand, opioid, benzodiazepine] or opioid monotherapy
Pharmacological therapy: Levodopa, benzodiazepines, and low-potency opioids [codeine, tramadol]	Pharmacological therapy: alpha-2-delta calcium channel ligands [gabapentin, pregabalin] and non-ergot dopamine agonists [pramipexole, ropinirole] if calcium channel ligands are contradicted	

Treating RLS in psychotic patients can be very challenging for many physicians. These challenges arise, as RLS is mainly treated by dopamine agonists, which work by mimicking dopamine activity [46]. Therefore, treating psychosis with antipsychotic medications, which inhibit dopaminergic neurotransmission, could exacerbate RLS symptoms [21-22]. Contrariwise, treating RLS symptoms in psychotic patients with dopamine agonists may lead to instability and frequent relapses of psychosis [47].

Olanzapine, Clozapine, and Quetiapine are the most frequently reported RLS-inducing antipsychotics [10]. However, there is a lack of clinical trials concerning RLS treatment in psychotic patients. Nevertheless, different approaches to the treatment of antipsychotic-induced RLS were followed in the cases reported in the literature. Several cases reported improvement of RLS symptoms after decreasing the dose of the antipsychotic used. Zhu et al. described a case of ziprasidone-induced RLS in a schizophrenic patient who scored 30 points on the International Restless Legs Syndrome Study Group Rating Scale (IRLS). This patient received ziprasidone 160 mg daily. A dose reduction to 120 mg daily resulted in the patient scoring nine on the IRLS [48]. Moreover, Rittmannsberger and Werl reported seven cases of Quetiapine-induced RLS, five of which were treated by the complete omission of Quetiapine, and one was treated by decreasing the Quetiapine dosage from 150 mg to 100 mg [36].

Additionally, other cases reported an alternative approach for treating antipsychotic-induced RLS involving a change in medication from one antipsychotic to another. For example, Olanzapine-induced RLS completely resolved after changing Olanzapine to aripiprazole, risperidone, and Quetiapine. Another report observed alleviation of RLS symptoms following the replacement of Olanzapine with Clozapine [11-13]. Due to its higher affinity to bind to D4 receptors than D2 receptors, Clozapine has a higher efficacy for the treatment of schizophrenia with fewer extrapyramidal symptoms; however, it is well established that clozapine might cause severe neutropenia and subsequent life-threatening agranulocytosis. Thus, baseline and regular monitoring of ANC is required [49-51]. In contrast, Duggal and Mendhekar reported complete remission of RLS symptoms in a patient with bipolar disorder treated with Clozapine after changing Clozapine to Olanzapine [51].

Furthermore, incorporating RLS medications into the initial antipsychotic regimen has demonstrated its efficacy in several cases. Hosoya et al. reported a case of treatment-resistant paranoid schizophrenia, which was successfully managed with 300 mg Clozapine and an RLS medication. A regimen of gabapentin enacarbil 600 mg/day and Clozapine 600 mg/day resulted in complete remission of RLS symptoms and good control of neuropsychiatric symptoms [52]. Additionally, Kumar and Venkatasubramanian reported two cases of Clozapine-induced RLS successfully treated after gabapentin was added to the regimen [10].

The combination of two or more of the previous approaches has also been proposed to successfully treat antipsychotics-induced RLS. Kang et al. treated a paranoid schizophrenic on Olanzapine 15 mg who scored 27 on the IRLS after initiating the treatment by adding benzodiazepines (Clonazepam 2 mg, Diazepam 5 mg, and zolpidem 10 mg). However, this only resulted in a mild improvement. Therefore, Olanzapine was reduced to 10 mg, and substantial improvement was observed afterward [14]. Nevertheless, it is of high importance to monitor signs of benzodiazepine physical dependency and addiction, such as drug-seeking behaviors, in such cases and intervene accordingly. 

## Conclusions

Antipsychotic-induced RLS is a widely accepted paradigm, with Olanzapine, Quetiapine, and Clozapine identified as the most common causes. However, the rationale behind the occurrence is poorly comprehended. The literature suggests that the development of RLS in antipsychotic users may be attributed to the inhibition of dopaminergic neurotransmission or the impact of antipsychotics on iron metabolism. Diagnosing antipsychotic-induced RLS remains a substantial challenge in clinical practice, with challenges in the management of this condition also being widely reported in the current literature. Further research is warranted to identify the underlying pathophysiology of antipsychotic-induced RLS and the genetic bases of this condition. This will enable clinicians to recognize those at risk of developing RLS and modify their antipsychotic regimen accordingly.
